# Prediction of Endocrine-Disrupting Chemicals Related to Estrogen, Androgen, and Thyroid Hormone (EAT) Modalities Using Transcriptomics Data and Machine Learning

**DOI:** 10.3390/toxics12080541

**Published:** 2024-07-26

**Authors:** Guillaume Ollitrault, Marco Marzo, Alessandra Roncaglioni, Emilio Benfenati, Enrico Mombelli, Olivier Taboureau

**Affiliations:** 1 Inserm U1133, CNRS UMR 8251, Université Paris Cité, 75013 Paris, France; guillaume.ollitrault@gmail.com; 2 Department of Environmental Health Sciences, Laboratory of Environmental Chemistry and Toxicology, Istituto di Ricerche Farmacologiche Mario Negri IRCCS, 20156 Milano, Italy; marco.marzo@marionegri.it (M.M.); alessandra.roncaglioni@marionegri.it (A.R.); emilio.benfenati@marionegri.it (E.B.); 3 Institut National de l’Environnement Industriel et des Risques (INERIS), 60550 Verneuil en Halatte, France; enrico.mombelli@ineris.fr

**Keywords:** endocrine-disrupting chemicals, gene expression, machine learning, estrogen, androgen, thyroid hormone, EAT

## Abstract

Endocrine-disrupting chemicals (EDCs) are chemicals that can interfere with homeostatic processes. They are a major concern for public health, and they can cause adverse long-term effects such as cancer, intellectual impairment, obesity, diabetes, and male infertility. The endocrine system is a complex machinery, with the estrogen (E), androgen (A), and thyroid hormone (T) modes of action being of major importance. In this context, the availability of in silico models for the rapid detection of hazardous chemicals is an effective contribution to toxicological assessments. We developed Qualitative Gene expression Activity Relationship (QGexAR) models to predict the propensities of chemically induced disruption of EAT modalities. We gathered gene expression profiles from the LINCS database tested on two cell lines, i.e., MCF7 (breast cancer) and A549 (adenocarcinomic human alveolar basal epithelial). We optimized our prediction protocol by testing different feature selection methods and classification algorithms, including CATBoost, XGBoost, Random Forest, SVM, Logistic regression, AutoKeras, TPOT, and deep learning models. For each EAT endpoint, the final prediction was made according to a consensus prediction as a function of the best model obtained for each cell line. With the available data, we were able to develop a predictive model for estrogen receptor and androgen receptor binding and thyroid hormone receptor antagonistic effects with a consensus balanced accuracy on a validation set ranging from 0.725 to 0.840. The importance of each predictive feature was further assessed to identify known genes and suggest new genes potentially involved in the mechanisms of action of EAT perturbation.

## 1. Introduction

Humans are exposed to various hazardous chemicals from diverse sources, such as the environment, diet, or medical treatment. Among all the chemical classes of xenobiotics, endocrine disrupting chemicals (EDCs) need special attention as they present peculiar properties due to their vulnerable windows of exposure and the long latency associated with the adverse effects they cause [1]. Evidence on the impact of EDCs on human health has considerably increased over the last few years, notably in cancer, metabolic disorders, neurocognitive functions, infertility, immune diseases, and allergies [2,3,4,5,6].

The epidemiological and toxicological knowledge on EDCs gathered since the pioneering work of Colborn et al. [7] that published an initial list of EDCs is currently accompanied by a study that provides an estimation of the economic impact that can be ascribed to the exposure of humans to EDCs [1]. According to this study, the estimated economic burden behind EDC-induced pathologies can be counted in hundreds of billions of euros per year.

The endocrine system is a complex machinery, and the estrogen, androgen, and thyroid hormone receptors (ER, AR, and TR)-mediated effects represent important nodes of this complex network. Indeed, many EDCs are known to target these receptor pathways [8,9]. ER and AR are critical regulators of reproductive functions (e.g., sex differentiation), whereas the thyroid axis presides over essential biological processes during neurodevelopment and the homeostasis of a normal physiological state. A direct binding to these receptors can result in various endocrine disruption effects impacting reproductive functions. However, reproductive functions can also be impaired by an effect on steroidogenesis, such as a perturbation of the aromatase enzyme, which is responsible for the conversion of androgens to estrogens. For this reason, a characterization of the perturbation of the ER, AR, and TR is usually accompanied by assays that can highlight the interference of chemicals with other enzymes involved in the thyroid pathway and with the steroidogenesis pathway [10].

Because of the special physiological importance of these biological pathways, their characterization with respect to endocrine disruption is regarded as crucial by many regulatory contexts, including the Tier 1 tests of the Endocrine Disruptor Screening Program of the US EPA [11,12], the level 2 and 3 mechanistic assays of the OECD framework for the testing and assessment of EDC [13], and the ECHA-EFSA guidance on the identification of EDCs related to biocidal products and plant protection products [14].

In addition, within the context of reduction, refinement, and replacement of animal use (3Rs), European and US directives support the move from traditional animal models to new approach methodologies (NAMs) in chemical risk assessment [15]. The National Research Council in the US outlined a general strategy for non-animal testing approaches, i.e., “Toxicity Testing in the 21^st^ Century in 2007 (Tox21)” [16], which has recommended including, among others, computational toxicology and in silico approaches in future assessments of toxicity as an inexpensive and efficient tool for screening purposes [17]. Such alternative methods, including integrated and computational approaches to testing and assessment (IATAs), allow evaluating large numbers of uncharacterized chemicals, including EDCs, while also reducing the time and cost of current approaches [13,16,17,18]. Therefore, many studies with a variety of in silico [19,20,21,22], in vitro [23,24,25,26], and in vivo methods [27,28] have been reported to detect EDCs.

Alongside in vitro and/or in vivo information, computational models (i.e., in silico models) can help in the assessment of human health hazards. More specifically, in silico studies can help prioritize chemicals for further testing. With the recent technological advances in EDC screening assays such as the Endocrine Disruptor Screening Program (EDSP) of the U.S. Environmental Protection Agency (USEPA) [29], in silico approaches, such as quantitative structure–activity relationships (QSARs), are largely considered for their ability to accurately predict toxicologically relevant endpoints [30]. However, they are exclusively related to the structure of the chemical and are based on the assumption that similar structures are associated with similar biological activity.

In this context, we conducted a computational study that considered the gene differentiation in two cell lines used in endocrine disruption studies, i.e., the MCF7 (breast cancer) and A549 (adenocarcinomic human alveolar basal epithelial) cell lines. The objective was to develop machine learning models using transcriptomics data associated with chemicals to predict the risk of EAT disruption. The principle is the same as a QSAR approach, i.e., chemicals sharing similar transcriptomic signatures (i.e., key genes) should share a similar toxicological behavior that can be described by QgexAR [31]. Together with predictive capabilities, another useful outcome of a QgexAR analysis is the possibility to suggest genes or metabolic pathways that can have predictive power (i.e., independent variables) and that can contribute to better characterizing the hazards of endocrine disruption.

In this article, a description and interpretation of EAT models resulting from different machine learning techniques, their potentiality, and their limitations will be discussed. The steroidogenesis model will not be performed because of the limited number of chemicals with transcriptomics data that are known to impact this endpoint.

## 2. Materials and Methods

### 2.1. Data

Various public datasets of chemicals covering the EAT pathway and general endocrine disruption effect were scrutinized with the aim of obtaining the largest possible set of chemicals for our analysis. We considered endocrine disruptor chemicals that trigger ER and AR adverse effects with agonist or antagonist effects on the receptors through binding, as suggested in the literature [32,33]. As far as the thyroid pathway is concerned, TR was considered.

#### 2.1.1. Estrogen Receptor (ER)

Chemicals capable of binding to ER were extracted from the Collaborative Estrogen Receptor Activity Prediction Project (CERAPP) [19]. CERAPP was a collaborative project aimed at showing the predictive potential of machine learning models trained on high-throughput screening (HTS) data to evaluate ER-related activity. To do so, CERAPP compiled a large dataset of HTS results for the ER. This dataset includes a training set of 1677 chemicals derived from U.S. EPA ToxCast™ and Tox21 in vitro data and an evaluation set of more than ~7000 chemicals compiled from multiple sources (HTS data from Tox21, the U.S. Food and Drug Administration (FDA) Estrogenic Activity Database, the METI database, and ChEMBL). 

#### 2.1.2. Androgen Receptor (AR)

Chemicals capable of binding to AR were extracted from the Collaborative Modeling Project for Androgen Receptor Activity (CoMPARA) [20]. Similar to the CERAPP initiative, CoMPARA was a collaborative project focused on demonstrating the predictive capabilities of machine learning trained on HTS data for assessing AR-related activity. CoMPARA compiled a large dataset of HRS data for the AR. This dataset includes a training set of 1662 chemicals derived from ToxCast™ and Tox21 in vitro data and an evaluation set of more than ~3800 chemicals compiled from PubChem [34].

#### 2.1.3. Thyroid Hormone Homeostasis

The homeostasis of the thyroid gland is a complex biological system. Datasets pertaining to the homeostasis of the thyroid gland were obtained from the research conducted by Gadaleta et al. [35]. The results of this research provide different datasets for different endpoints related to thyroid hormone pathways while covering several protein families related to thyroid perturbation, i.e., DIO (deiodinases 1, 2, and 3), TPO (thyroid peroxidase), TR, NIS (sodium iodide symporter), TRHR (thyrotropin-releasing hormone receptor), and TSHR (thyroid stimulating hormone receptor). The datasets are compiled from the ToxCast™ program (DIO, TPO, and NIS) and the Tox21 program (TR, TRHR, TSHRAnt, and TSHRAg). The TPO and TR endpoints are described as the most important ones [35]. As there was too little data for the TPO endpoint, only the TR antagonist endpoint could be modeled. In this respect, TR data from the assay “antagonistic modulation of TR antagonist measured via thyroid hormone-dependent luciferase expression” were adopted for modeling (6342 chemicals).

### 2.2. Transcriptomic Data

Within the collection of chemicals described above, comprising experimental information on ER, AR, and TR, only chemicals that could also be associated with information on transcriptomics were considered for further analysis. Transcriptomic data were retrieved from the LINCS L1000 project. The project experimentally measured 978 universally informative transcripts, which they termed “Landmark Genes”. The remaining genes of the transcriptome were then inferred according to these measurements. Of the 11,350 inferred genes, 9196 were said to be “best inferred” as they displayed good gene-level recall performance > 0.95. The remaining 2154 genes with a lower recall were said to be “inferred” and removed from this analysis. The database consists of over 1.3 million transcriptomics gene expression profiles for 34,419 chemicals [36].

Our analysis focused on cell lines that had been previously reported for EDC analysis, i.e., MCF7 (breast cancer) [37] and A549 (adenocarcinomic human alveolar basal epithelial) [38,39] cell lines. The majority of the chemicals have been tested at 24 h and 10 μM and are considered in our analysis. Each chemical has a measurement of differential expression (determined as a z-score) for each of the 12,327 genes. 

### 2.3. Dataset Development

From the set of chemicals with established EAT activities, multiple datasets were derived. Each of them was associated with transcriptomic profiles specific to different cell lines. The number of active and inactive chemicals corresponded to the annotations collected from the datasets above for each endpoint and cell line (Table 1).

#### 2.3.1. Data Preprocessing and Standardization

All the chemicals were mapped to their PubChem chemical ID (CID) in order to have standardized chemical structures according to the PubChem standardization protocol [9] (i.e., normalization of the representation, implicit hydrogen atom valence, tautomeric form representation, etc.). Chemical mixtures were not considered in the analysis. The PubChem CID was retrieved according to the available SMILES, CAS RN, name, and InChI available from the source databases. This standardization allowed for the removal of duplicate chemicals. In cases of duplicates with contradicting experimental evidence, the chemical was considered active. In cases of duplicate gene expression profiles for the same chemical in the LINCS, the profile with the highest transcriptional activity score (TAS) was retained, as described by Subramanian et al. [36].

#### 2.3.2. Biological Features

Differential gene expression signatures for a total of 12,328 genes with 978 biologically tested genes (defined as landmarks), 9196 inferred genes with a high confidence score (defined as gene level recall R_gene_ > 0.95 and called best inferred), and 2154 inferred genes with a lower confidence are defined as features. The gene signatures are computed using z-scores (continuous values). A negative z-score describes a down-regulation of gene expression (negative differentiation) caused by the chemical, while a positive z-score represents an up-regulation of gene expression (positive differentiation) induced by exposure to a chemical. 

### 2.4. Machine Learning Algorithms

Several machine learning algorithms, automatic machine learning protocols, and deep learning models were tested, including CATBoost [40], XGBoost [41], Random Forest [42], SVM (SGDClassifier) [43], Logistic regression (Elastic Net) [44], AutoKeras [45], TPOT [46], and deep learning models (four fully connected neural network models).

− Logistic regression is a statistical method that models an activity as a linear combination of input variables by applying a logistic function to the result.
− Support Vector Machines (SVMs) found the hyperplane that maximizes the margin between two classes in feature space and classifies new points based on which side of the hyperplane they belong to.
− Random Forest (RF) is an ensemble learning method that combines the output of multiple unpruned decision trees to make a prediction.
− XGBoost is an optimized gradient boosting library built to implement highly efficient parallel tree boosting models where each subsequent tree attempts to correct the errors made by the previous tree. CATBoost is another optimized gradient boosting library that uses ordered boosting, and is designed to handle categorical data.
− A deep neural network (dnn) is a type of artificial neural network composed of nodes (“neurons”) gathered into layers. Dnns are composed of multiple hidden layers connected to each other, where information is passed between layers and serves to update the function (ReLU) in each node of the network in order to minimize the prediction error.
− AutoKeras is an automated machine learning (AutoML) library based on Keras [47] that automates the process of building deep learning models.
− TPOT is a Python AutoML tool that optimizes machine learning pipelines using genetic programming. All machine learning models and preprocessing steps are part of Scikit-learn [48] library.

Hyperparameter optimization was performed for the different models (Table S1). Balanced weight was considered in the XGBoost, CATBoost, RF, SVM, and Logistic regression models. These models’ parameters were optimized using grid search with 5-fold cross-validation using the balanced accuracy scoring method.

### 2.5. Dataset Preparation

The datasets are split into a training set and a validation set. The validation set is developed by randomly sampling a subset of chemicals present in all the two cell lines for each endpoint. The distribution of the chemicals in the training set and the validation are shown in Figure S1.

The training set was used to develop, optimize, and select the best-performing algorithm. No information from validation sets was used for selecting and optimizing models.

An inner 5-fold cross validation (for hyperparameter optimization with grid search) was performed for CATBoost, XGBoost, SVM, Logistic regression, Random Forest, and TPOT. Then, an outer repeated 10 times 5-fold cross validation was applied for model selection and performance assessment on all the models on the training set. Balanced accuracy (defined in the following paragraphs) was used to identify the optimal model parameters and also during the application of the protocol for feature selection.

The final best models were used to evaluate the predictive performance of the unseen chemicals belonging to the validation datasets.

#### 2.5.1. ER Binding

Chemicals with known ER binding activity were selected using the training set and validation set from CERAPP. Subsequently, we cross-referenced these selections against transcriptomics data. The cell lines MCF7 and A549 yielded 1552 and 1262 chemicals (Table 1), respectively, with available transcriptomics data. Among these, a total of 1259 chemicals were found to be common across the two cell lines. From this common set, a random selection of 291 chemicals was made to form the validation set, comprising 100 active and 191 inactive chemicals (Table 2).

#### 2.5.2. AR Binding

Chemicals with known AR binding activity were selected using the training set and validation set from CoMPARA. The cell lines MCF7 and A549 yielded 1000 and 804 chemicals, respectively (Table 1), with available transcriptomics data. Among these, a total of 802 chemicals were found to be common across all four cell lines. From this common set, a random selection of 200 chemicals was made to constitute the validation set, comprising 45 active and 155 inactive chemicals (Table 2).

#### 2.5.3. TR Antagonistic Mode of Action 

Transcriptomics data for the MCF7 and A549 cell lines yielded 1236 and 100 chemicals, respectively, for the TR analysis. Among these, a total of 998 chemicals were found to be common across the two cell lines. A random selection of 150 chemicals was made to constitute the validation set, comprising 56 active and 94 inactive chemicals that were common to all five cell lines (Table 2).

### 2.6. Feature Selection

In order to reduce the number of features and increase the parsimony and interpretability of the models, the multiSURF method was applied to filter and retain only the most informative features [49]. The algorithm selects features and assigns positive and negative weights by searching for value differences between nearest neighbor elements having different activities. The multiSURF algorithm adopts a distance threshold *T* as the mean pairwise distance between the target instance and all others to determine which instances will be considered neighbors. It also introduces a dead band zone to identify instances that are ambiguously near or far neighbors. The selection of the features was performed exclusively on the training sets. Any features with a score superior to 0 were retained [49].

### 2.7. Distribution of Chemicals in a Transcriptomic Space

To visualize the distribution of chemicals in a 2D space with respect to their transcriptomic profile, a UMAP representation was generated with Python, and the UMAP package was developed [50]. For the representation, we considered the landmark genes. The resulting visualization was plotted using Plotly [51], facilitating a comprehensive exploration of the transcriptomics landscape and enabling insightful analysis of the distribution of chemicals according to the cell lines and the endpoint activity.

### 2.8. Predictive Performance

The predictive performance of the models on the training sets was assessed using 10 iterations of 5-fold cross-validation. The cross-validation procedure was performed on the different models by computing sensitivity, specificity, balanced accuracy, the Matthews correlation coefficient (MCC) [52], and area under curve (AUC). These metrics vary between 0 and 1, with a value of 1 indicating high performance.

In the second step, the models were evaluated on a validation set (data were not used for calibrating, optimizing, and/or selecting models), and the predictive performance was evaluated using the same statistical indicators. 

The adopted performance metrics are defined as follows:

(1) $$\mathrm{Sensitivity}\;(\mathrm{SEN})=\frac{TP}{TP+FN}$$

(2) $$\mathrm{Specificity}\;(\mathrm{SPE})=\frac{TN}{TN+FP}$$

(3) $$\mathrm{Balanced}\;\mathrm{accuracy}\;(\mathrm{BA})=\frac{\mathrm{Sensitivity}\;+\;\mathrm{Specificity}}{2}$$

(4) $$\mathrm{MCC}=\frac{TP\times TN\;–\;FP\times FN}{\sqrt{\left(TP+FP\right)\left(TP+FN\right)\left(TN+FP\right)\left(TN+FP\right)}}$$

(5) $$\mathrm{AUC}=\int_{x=0}^{1}\frac{\mathrm{Sensitivity}}{\left(1-\mathrm{Specificity}\right)\left(x\right)}dx$$

where *TP* stands for true positive, *TN* for true negative, *FN* for false negative, and *FP* for false positive. The AUC quantifies the discriminative capability of a binary classification model across various classification thresholds, ranging from 0 to 1.

The mean value of each metric was computed when analyzing the results provided by cross-validation.

Most importantly, the mean BA of cross-validation was used as a metric to select the best-performing hyperparameters and models since it is well suited for data imbalance [53] (the majority of inactive chemicals in the context of this article), while considering sensitivity and specificity to better evaluate the detection of active chemicals even when there are few positive examples. 

### 2.9. Consensus Prediction

The developed models for the two cell lines using the best machine learning model result in two predictions exploring different gene expression profiles on different cell targets. Some chemicals could act differently on different cell lines and thus be well predicted in most cell lines and badly predicted on a few others. In order to overcome these limitations, a consensus prediction was defined by considering the best feature selection protocol and the best machine learning algorithm in cross-validation by taking the majority prediction across the two models for each cell line. The consensus prediction was set to be the mean probability prediction of the models of the 2 cell lines. The consensus prediction was evaluated on the validation set shared by the two cell lines. 

### 2.10. Feature Importance

The importance of the predictive features was assessed based on the Kruskal–Wallis non-parametric test, as the features do not follow a normal distribution. The genes with a *p*-value inferior to 0.05 were counted. We further explored links with ED of these top 10 differentially expressed genes, showing the lowest *p*-values common to multiple cell lines between active and inactive chemicals for each endpoint.

### 2.11. Applicability Domain

The applicability domain is a concept utilized in QSAR modeling [54] to denote the model’s ability to accurately predict outcomes for unseen data, thus reflecting its overall quality and defining the feature space within which a model can yield predictions with a given reliability. It can prevent misuse of the model and increase the reliability of a given prediction. It is defined in the training set of the model and serves as a criterion to state for which chemicals the model can be reliably applicable. 

In this study, we opted for a distance-based approach that relies on assessing the similarity between the transcriptomics profile of a query chemical and those in the training set. This similarity was computed using the cosine similarity metric. We computed the mean cosine similarity score of the three most similar chemicals from the training set for each query of the validation set. The performance of the models was then compared by evaluating their precision across various threshold values.

Defining a precise threshold to determine whether a chemical is within or outside the applicability domain is a compromise between precision and coverage of the chemical space.

Several approaches exist for setting a threshold, and we chose to define it as Dc = <y> − Zσ. Here, <y> represents the average, and σ is the standard deviation of the cosine similarity score of the three nearest training set neighbors of each chemical in the training set. The parameter Z controls the significance level, with a default value of 0.5. The key difference with the original formula is in adjusting <y> by subtracting Zσ, as we utilize a cosine similarity score ranging from 0 to 1, where 1 indicates similar transcriptomics profiles, whereas the original formula adopts Euclidean distance, where 0 indicates a similarity [55,56].

### 2.12. Protocol

**Figure 1:**
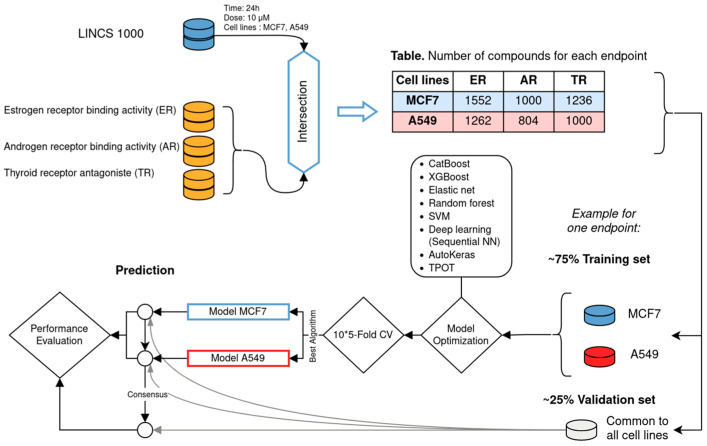
Protocol for the development and evaluation of predictive models using transcriptomics data. Endocrine disruptors activity related to ER, AR and TR were retrieved from CERAPP [19], CoMPARA [20], and Gadaleta et al. [35] respectively. Transcriptomics data were retrieved from L1000 [36].

The protocol for the development and evaluation of the models of EAT-related modalities is illustrated in Figure 1. The transcriptomics data and the EAT biological data are combined for each endpoint and cell line. Each dataset is split into a training set and a validation set, which are common to the two cell lines. Models are optimized using the training set and evaluated through 10 iterations of 5-fold cross-validation. The optimized model is then trained on the entire training set, and its performance is evaluated on the respective validation set.

## 3. Results

### 3.1. Transcriptional Space

**Figure 2:**
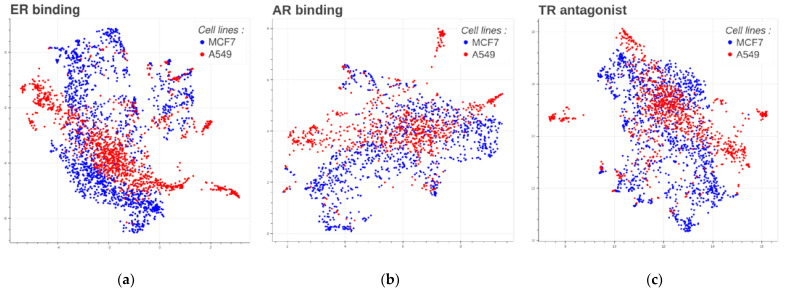
UMAP of the transcriptomics profiles for each chemical in each dataset for the two cell lines. Each dot corresponds to a chemical. Blue dots correspond to chemicals in the MCF7 dataset and red to A549. Only landmark genes were considered. (**a**) Profile for the ER binding dataset. (**b**) Profile for the AR binding dataset. (**c**) Profile for the TR antagonist dataset.

The ER binding, AR binding, and TR antagonist datasets reveal significant transcriptional diversity among different cell lines (Figure 2). Chemicals display distinct transcriptomics profiles across cell lines, which can be explained by the fact that some genes are only expressed in specific cell lines. This diversity offers an opportunity to investigate various effects that a chemical may exert on different cell lines, tissues, and organs, thereby providing some hypotheses on the mechanisms of action perturbed by a chemical at the molecular level (genes) and in relation to a specific endpoint (i.e., endocrine disruption). Such an approach enhances our ability to comprehend the multifaceted effects of chemicals and their potential implications across various biological contexts.

**Figure 3:**
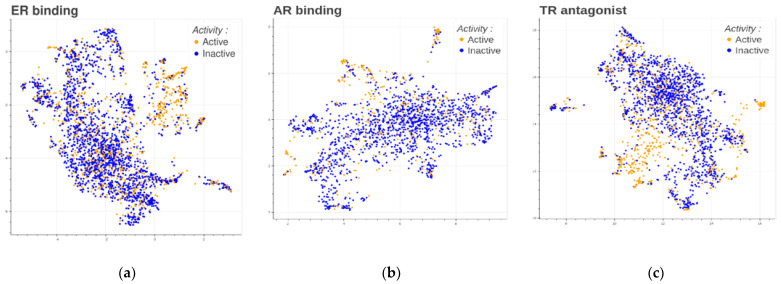
UMAP of the transcriptomics profiles using the landmark genes for each chemical in each dataset for the two cell lines. In orange are the active chemicals, and in blue are the inactive chemicals, considering the specific endpoint. (**a**) Profile for the ER binding dataset. (**b**) Profile for the AR binding dataset. (**c**) Profile for the TR antagonist dataset.

Figure 3 displays the relationship between groups of chemicals (active/inactive) and their corresponding gene expression profile relationships. The plots illustrate that some active chemicals (orange dots in the upper part of the plots) have a global gene expression relatively different from that of inactive chemicals (blue dots).

This connection by clustering close groups of points within the same regions shows the similarity in their gene expression patterns and activity profiles. The observed patterns and clustering patterns in the plot justify the use of gene expression profiles as features for predicting EAT modalities. Machine learning models can effectively differentiate between active and inactive chemicals by leveraging close groups of chemicals sharing similar gene expression profiles.

### 3.2. Protocol Selection for Cross-Validation

In this study, we trained ten machine learning models (CATBoost, XGBoost, RF, elasticnet, rf, SVM, dnn_1, dnn_2, dnn_3, dnn_4, dnn_autokeras, and TPOT) on transcriptomics data for two cell lines (MCF7 and A549) in order to predict the activity of chemicals on three endpoints covering EAT endocrine disruption modalities, i.e., ER binding, AR binding, and TR antagonist. Due to the large number (12,328 genes) of features and their potential uncertainty (inferred genes), we tested multiple feature subsets with and without feature selection with the multiSURF algorithm (Table S2). The performance of the models was evaluated by 10 iterations of 5-fold cross-validation. 

The results of QGxAR modeling are shown in Table S2. One of the most evident characteristics of the different ER, AR, and TR models is that models based on landmarks outperformed models including inferred genes. The second observation is that models trained on landmark genes after feature selection through multiSURF showed, in general, a small but significant improvement (*t*-test *p*-value of 0.013 < 0.05) compared to models solely trained on landmark features (Table S2). Therefore, landmark genes seem to capture most of the biological information, and the inferred genes introduce extra noise. The implementation of a selection algorithm reduces the number of features to learn from, retaining those that have the strongest impact on enabling a prediction of the endpoint of interest. In this respect, starting from the initial 978 landmark genes, feature selection reduced the number, ranging from 72 to 80 genes selected across the three endpoints and the two cell lines.

**Figure 4:**
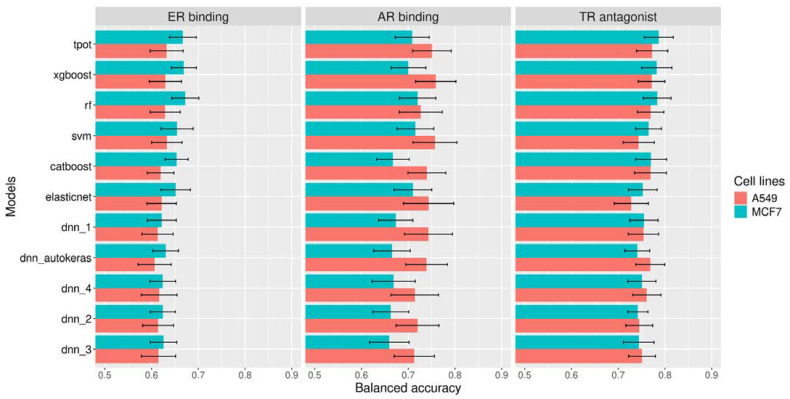
Mean BA from cross-validation (BA-CV) with standard deviation (BA-CV sd) after 10 iterations of five-fold cross-validation on the training set for all the cell lines and all the models for all the endpoint datasets using the landmark features selected by the multiSURF algorithm.

In terms of BA during cross-validation on the training set for the ER binding endpoint, the optimal feature selection method consisted of using only the landmark genes in conjunction with the multiSURF selection algorithm. For the ER binding endpoint in all the developed consensus models (MCF7 and A549 cell lines), the best performance was obtained with the RF algorithm (Figure 4), with a BA ranging from 0.629 to 0.672. The mean sensitivity for this endpoint in these models was low, with values ranging from 0.413 to 0.482. The low sensitivity could be attributed to the imbalanced dataset. This is the reason why we chose to select the best model based on BA, as it is best suited for imbalanced datasets.

For the AR binding endpoint, when considering the landmark feature with the multiSURF method for feature selection, the best performance between the two cell lines in terms of BA was obtained with the SVM algorithm, with a BA across the two cell lines ranging from 0.715 to 0.757 (Table S2), with the model on A549 performing the best and the model on MCF7 performing the worst. For TR antagonists, the optimal feature selection method consisted of only the landmark genes in conjunction with the multiSURF selection algorithm. The best performance in terms of BA was obtained with the TPOT method, with BA across the two cell lines ranging from 0.773 to 0.787 (Table S2). The performance of the MCF7 model was better compared to the A549 model.

### 3.3. Performance of the Prediction

Subsequently, we evaluated the prediction performance on a validation set and developed a consensus prediction by considering the probability of prediction from the two best models for the two cell lines.

We selected landmark features based on them and further refined the feature selection using the multiSURF algorithm (Table 3). The best cross-validation performance was achieved using the Random Forest method for the ER binding, TPOT for the TR antagonist endpoints, and SVM for the AR binding endpoint. We evaluated the models on a separate validation set that was not used for training, and we obtained good performance, showing that our models were robust, rather accurate, and in three cases very accurate (i.e., BA > 0.8), being able to predict the hazard for a new chemical to lead to endocrine disruption. More specifically, for ER binding, the best validation performance was achieved on the MCF7 cell line, with a BA of 0.713. For AR binding, the highest validation performance was observed on the A549 cell line, with a BA of 0.804. Lastly, for the TR antagonist endpoint, the most favorable results were obtained on the MCF7 cell line, with a BA of 0.824.

By considering the two QGexAR models for each cell line, a consensus prediction was obtained by averaging the probabilities of being an ER binder, an AR binder, or a TR antagonist. The consensus method reached a BA of 0.725, 0.763, and 0.840 on the validation set for ER binding, AR binding, and TR antagonist, respectively. The consensus prediction improved performance in terms of BA for the ER binding and TR antagonist endpoints. For the ER binding, the increase in BA also reduced the SE. For the AR binding endpoint, the consensus prediction performed lower than the model on A549. These results show that some chemicals are better predicted for specific cell lines, and a consensus model can, in some endpoints, increase predictive performance, thus taking advantage of the effect of chemicals on different cell lines.

### 3.4. Comparison with Already-Published Models

The predictive performance of our QGexAR models on the validation set using the consensus models with respect to ER binding and AR binding was compared to the QSAR models developed by the CERAPP and CoMPARA projects (Table 4). All chemicals in our evaluation set were predicted using the QSAR ER binding and AR binding models of CERAPP and CoMPARA. 

In the CERAPP and CoMPARA projects, the authors noticed that with the increase in the number of sources in the literature, data can provide information about the repeatability of the results and thus about their accuracy. In CERAPP and CoMPARA, they used different literature evidence scores. Considering a threshold of one piece of literature evidence (i.e., activity reported in at least two publications), we compared the predictive performance of our models to the CERAPP and CoMPARA projects. The evaluation set composed of 291 chemicals was reduced to 231 chemicals with 51 active and 180 inactive for the ER binding endpoint and was reduced from 200 to 101 chemicals with 17 active and 84 for the AR binding endpoint after applying the literature threshold.

The QGexAR ER binding consensus model resulted in higher performance than the QSAR model of CERAPP, either on all our evaluation sets or considering only high-confidence chemicals. The BA for all the evaluation sets was 0.725 for the QGexAR ER binding model and 0.615 for the QSAR ER binding model. Similarly, the BA for the set with high confidence data was 0.827 for the QGexAR ER binding model and 0.687 for the QSAR ER binding model.

However, the QGexAR AR binding consensus model showed lower performance than the CoMPARA QSAR model across our evaluation set yet displayed superior performance when evaluating only high-confidence biological activity chemicals within our dataset. The BA for all the evaluation sets was 0.807 for the QGexAR AR binding model and 0.778 for the QSAR AR binding model. For the evaluation set with high-confidence data, the BA was 0.888 for the QGexAR AR binding model and 0.763 for the QSAR AR binding model. 

It is important to note that some chemicals in our evaluation set could have been used during the learning process of the CERAPP and CoMPARA QSAR models, which suggests an overestimation of the QSAR model’s performance compared to QGexAR. Hence, QGexAR models for ER and AR binding performed reasonably well for high-quality data supported by multiple literature sources.

The QGexAR ER binding consensus model accurately predicted 54 chemicals that were poorly predicted by the QSAR ER model, while the QSAR ER model effectively predicted 34 chemicals that were poorly predicted by the QGexAR ER binding consensus model. Similarly, the QGexAR AR binding consensus model successfully predicted 48 chemicals that were inadequately predicted by the QSAR AR model, while the QSAR AR model effectively predicted 21 chemicals that were poorly predicted by the QGexAR AR binding consensus model. These findings highlight the complementary nature of both approaches and their respective strengths and weaknesses.

As the model for TR antagonists outlined by Garcia de Lomana et al. [57] was inaccessible, our comparison was limited to utilizing the BA reported within their publication. Despite this constraint, their model demonstrated good performance, achieving a BA of 0.80. In our study, our model exhibited comparable performance, achieving a BA of 0.84.

### 3.5. Feature Importance

We analyzed differentially expressed genes between active and inactive chemicals for the different endpoints using the Kruskal–Wallis statistic test. The most significant genes in terms of the lowest *p*-value (<0.05) for each cell line were further analyzed to identify known associations with endocrine disruption in the scientific literature. Genes that were not reported in the literature but highlighted by our work can be further studied for their possible mechanisms regarding endocrine disruption.

For the ER binding endpoint, the number of significant genes showing a gene expression difference between active and inactive chemicals ranges from 3267 (A549 cell line) to 4812 (MCF7 cell line) genes for a *p*-value threshold of 0.05. Among the 10 genes that are significantly differentially expressed between ER binder and non-ER binder, we identified the *DDIT4*, *CCNA2*, *CDCA3*, *MCM10*, and *TOP2A* genes. 

*DDIT4* is a gene enabling protein binding activity; it is found to be overexpressed (included in the top 10 genes) in MCF7 cell lines in active chemicals. Its link with endocrine disruption has been observed in the literature, as phthalate chemicals considered endocrine receptors were reported to upregulate *DDIT4* in MCF7 cell lines [58]. *DDIT4* was also found to be upregulated by two endocrine disruptors, i.e., bisphenol A and bisphenol S [59]. CCNA2 encodes proteins involved in the regulation of the cell cycle. It is underexpressed (top 10) in the MCF7 cell lines. CCNA2 was found to contribute to prostate cancer through the modulation of its expression [60]. MCM10 encodes a mini-chromosome maintenance protein involved in the initiation of eukaryotic genome replication. It is underexpressed (included in the top 10 genes) in the MCF7 cell lines. It was shown that the expression level of MCM10 was higher in breast cancer cell lines [61]. *TOP2A* encodes a DNA topoisomerase. It is underexpressed (included in the top 10 genes) in the A549 cell lines as an active chemical. *TOP2A* was shown to be prognostic in estrogen receptor-positive breast carcinomas [62].

For the AR binding endpoint, the number of significant genes showing a gene expression difference between active and inactive chemicals ranges from 3233 (MCF7 cell line) to 4507 (A549 cell line) genes for a *p*-value threshold of 0.05. Among the 10 genes that are significantly differentially expressed between AR binder and non-AR binder, we found the *DDIT4* and MCM10 genes. *DDIT4* and MCM10 were also found to have ER binding among the top 10 MCF7 cell lines. 

For the TR antagonist endpoint, the number of significant genes showing a gene expression difference between active and inactive chemicals ranges from 4257 (A549 cell line) to 5474 (MCF7 cell line) genes for a *p*-value threshold of 0.05. Among the 10 genes that are significantly differentially expressed between TR antagonists and non-TR antagonists, we found genes that showed evidence of links with endocrine disruption in the literature, i.e., DDIT4 [59], *SQSTM1* [63], *CTSL* [64], and TOP2A [65]. The genes *CDCA3* and *SPC25* were significantly differentially expressed in MCF7, but no links with endocrine disruptors could be found in the literature. These genes could be further explored to decipher the possible mechanism that links them with endocrine disruption.

The percentage of differentially expressed genes among the best inferred and landmark genes between active and inactive chemicals across endpoints ranges from 32 to 54%. In our models, the application of a selection method involving landmark genes along with multiSURF analysis allowed us to retain 55 to 91% of these genes with significant differentially expressed values (Table S3). This highlights the pertinence of this selection method in capturing the most important genes for the development of machine learning models. However, landmark genes are only a small portion of the gene space, consequently reducing the performance of our models, as some genes could be of more importance but for which there is no tested biological data and only inferred values.

### 3.6. Applicability Domain

**Figure 5:**
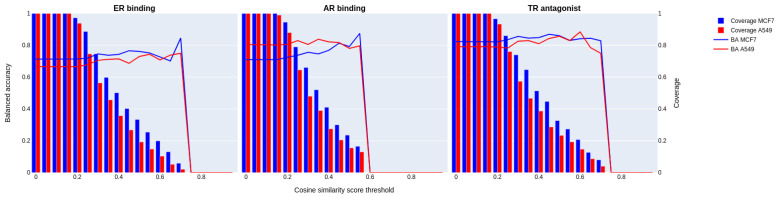
Predictive performance of the top-performing models retained for each cell line across the three endpoints on the validation set was determined by varying the cosine similarity thresholds from 0 (not similar) to 1 (similar). The *x*-axis denotes the cosine similarity threshold utilized for inclusion of the transcriptomics profile of the validation set in the performance calculation. Cosine similarity is determined by averaging the similarity scores of the three most similar transcriptomic profiles, utilizing landmark and best-inferred genes. The solid lines indicate BA, whereas the bars represent the coverage of the validation set.

The applicability domain was explored for the three endpoints using a three-nearest neighbor method on the validation set. For each chemical from the validation, we computed the cosine similarity of its transcriptomics profile considering the landmarks and best inferred genes vs. all the transcriptomics profiles of all the chemicals in the training set. We computed the mean cosine similarity of the three chemicals closest neighbors. We considered cosine similarity thresholds ranging from 0 to 1. The evolution of the predictive performance was monitored for each endpoint and each cell line, as was the coverage of the validation set (Figure 5).

As the applicability domain narrows and the validation set comprises more chemicals with transcriptomics profiles similar to those of the training set, our models exhibit enhanced performance for the three endpoints and two cell lines. We note some disparities in performance evolution between cell lines. For the ER binding endpoint, the models developed as a function of the MCF7 cell line have better performance than the model developed for the A549 cell line. For AR binding, the A549 model has better performance than the model for the MCF7 cell line. For the TR antagonists, the model for the MCF7 cell line has better performance than the model developed for the A549 cell line. The predictive performance for all models developed with the different cell lines first increases and then decreases rapidly when the threshold is high. 

For the different endpoints and cell lines, we observe that the model’s performance stops increasing and falls to 0 around a cosine similarity threshold of 0.4–0.5. 

This behavior is due to the reduced number of chemicals used for performance evaluation. When the number of chemicals used to evaluate performance is close to 0, the robustness of performance metrics is largely challenged. The presence of a slightly lower performance when increasing the threshold (cosine similarity) could also be attributed to the presence of chemicals that are inaccurately predicted, even in the presence of similar transcriptomics profiles within the training set (i.e., similarity paradoxes). For example, the chemical Eritrocina, present in the validation set of the ER binding endpoint, is predicted incorrectly as inactive even though it has a high mean cosine similarity of 0.682 with its three closest neighbors.

We explored the performance of the models after setting a cosine similarity threshold (removing chemicals that are outside of the applicability domain). The threshold was considered using the formula Dc = <y> − Z*sigma. We computed the performance based on the validation for the set of chemicals retained after applying this threshold. The results are presented in Table 5. After applying the applicability domain to the three endpoints, we noticed increased performance for the ER binding endpoints for all cell line models. For the AR binding endpoint, the performance is increased for all the cell line models. For example, for the A549 model, the BA is increased from 0.804 to 0.806. For the TR antagonist endpoint, the performance is increased for all the cell line models. For example, in the MCF7 model, the BA is increased from 0.824 to 0.845. Applying the applicability domain reduced the coverage for the different models; coverage was still close to or superior to 50% and ranged from 48 to 65.3%.

## 4. Discussion

The underlying paradigm of QSAR models that can be used for prioritizing chemical lists for further testing involves the use of information on chemical structure to predict biological effects, with the assumption that similar chemicals exhibit similar biological activities. However, this approach can have some limitations, notably in toxicity, due to the fact that the chemical space might be quite large and different mechanisms of action can lead to the same adverse outcome. Many chemicals with low structural similarity might trigger the same biological pathways. With QGExAR models, instead of relying on chemical structures, the gene expression profiles of two different cell lines exposed to a set of chemicals experimentally known as AR binders, ER binders, and TR antagonists were used to develop machine learning QGExAR models.

Transcriptomics allows us to observe the effects of a chemical at the cellular level through the expression of genes. New and fast experimental methodologies [36,66] have enabled the screening of a large number of chemicals and provided a deeper mechanistic understanding of the effects of these chemicals, facilitating the development of machine learning models to predict the toxicity of untested chemicals.

In LINCS, the gene expression profiles typically cover only a small set of genes (978 landmark genes), with the rest being predicted. We have observed that using only landmark genes improves the predictability of our models. The use of predicted profiles can introduce biases into the models and their predictions [67].

The analysis of landmark genes, best-inferred genes, and inferred genes revealed distinct clustering patterns. Inferred genes tended to group separately from both landmark and best-inferred genes in the feature space (Figure S2), raising concerns about their potential to mislead predictive models and diminish performance.

Furthermore, by exclusively selecting landmark genes, we might overlook other highly informative genes that were only predicted, like the ER-related genes (ESR1 and ESR2), the AR gene, and the thyroid hormone receptor genes (THRA and THRB). We are aware that using only landmark features (978 genes) reduces the multi-faceted reality of gene differentiation in a cell line, and developing machine learning models on them might hinder some mechanisms not represented by other sets of genes. Neglecting these potentially important genes could lead to biased or incomplete models, and caution has to be considered in the interpretation of the mode of action regarding the specific endpoints. Nevertheless, these landmark genes have been selected because they play key roles in the biological processes of a cell. Therefore, they can provide the first information source to look at for estimating the hazards of endocrine disruption.

A limitation of our work is that we considered only one concentration and one time, as it was the one for which we had the most chemicals with transcriptomics data. It is worth noting that a chemical might exhibit different effects at different doses and/or with prolonged exposure. Also, our work focused on two cancer cell lines, i.e., MCF7 and A549, used in endocrine disruption studies, for which we have enough chemicals to perform our in silico analysis. A similar approach could be developed in the future on the H295R cell line for steroidogenesis or in stromal and immune cell types in tumor environments (TMEs), for which endocrine disruption studies have been reported [68,69]. A QGExAR analysis on a large set of cell lines (normal, primary, differentiated, etc.) would be of interest to characterize specific gene deregulation and the dedicated mechanisms of action that drive endocrine disruption by groups of chemicals.

## 5. Conclusions

With the emergence of machine learning for predicting the toxicity of chemicals, the development of QGexAR models is gaining prominence. This approach, in addition to being an efficient method and complementary to widespread QSAR modeling, allows us to explore the mechanisms of chemicals in relation to their toxicity. Our results show that we were able to develop robust and predictive QGexAR models covering EAT modalities for endocrine disruption. Our models considered multiple cell lines, i.e., MCF7 and A549, providing us with a wide array of differentially expressed genes in different cell lines when exposed to chemicals. We successfully identified the most relevant features from our models, which we used to train machine learning algorithms, and by considering two cell lines and the associated consensus prediction, we characterized robust models.

The developed QGexAR models can contribute to the development of safer chemicals in relation to endocrine disruption and to the prioritization of chemical lists for further toxicological investigations. However, more data are still needed to comprehensively address, for example, the steroidogenesis pathway and all the receptors of the thyroid pathway.

## Figures and Tables

**Table 1:** Adopted datasets for QgexAR modeling. Information on the biological response of human cell lines was retrieved from the LINCS program. The number of chemicals present in both cell lines is described in “MCF7 ∩ A549”.

Cell Line	Chemicals	ER	AR	TR
MCF7	Total	1552	1000	1236
	Active	484	215	404
	Inactive	1068	785	832
A549	Total	1262	804	1000
	Active	424	193	359
	Inactive	838	611	641
MCF7 ∩ A549	Total	1259	802	998

**Table 2:** Training and validation sets adopted for developing QgexAR models. The validation set (MCF7 and A549) is a set composed of chemicals present in the two cell lines.

Cell Lines	Dataset	Chemicals	ER	AR	TR
MCF7	Training	Total	1261	800	1086
		Active	384	170	348
		Inactive	877	630	738
A549	Training	Total	971	604	850
		Active	324	148	303
		Inactive	647	456	547
MCF7 ∩ A549	Validation	Total	291	200	150
		Active	100	45	56
		Inactive	191	155	94

**Table 3:** Predictive performance for the binding, AR binding, and TR antagonist considering landmark features with multiSURF-selected subsets of predictive features. Performance was evaluated on the validation set in common between the MCF7 and A549 cell lines, and the consensus performance on the validation set is computed by averaging the probability of prediction from the two models for the MCF7 and A549 cell lines. The consensus prediction cannot be computed on the training set as there are different numbers of chemicals between the training sets of the two cell lines, which was noted as “NA”. Sensitivity (SE); specificity (SP); balanced accuracy (BA); Matthews coefficient correlation (MCC); and area under curve (AUC).

Cell Line		Training					Validation				
	Best Algorithm	SE	SP	BA	MCC	AUC	SE	SP	BA	MCC	AUC
ER binding											
MCF7	RF	0.878	0.937	0.907	0.810	0.907	0.610	0.817	0.713	0.431	0.713
A549	RF	0.898	0.941	0.920	0.836	0.920	0.540	0.791	0.665	0.336	0.665
Consensus		NA	NA	NA	NA	NA	0.570	0.880	0.725	0.478	0.764
AR binding											
MCF7	SVM	0.824	0.806	0.815	0.549	0.815	0.711	0.710	0.710	0.361	0.710
A549	SVM	0.899	0.816	0.857	0.640	0.857	0.867	0.742	0.804	0.520	0.804
Consensus		NA	NA	NA	NA	NA	0.622	0.903	0.763	0.534	0.846
TR antagonist											
MCF7	TPOT	0.730	0.888	0.809	0.623	0.809	0.786	0.862	0.824	0.645	0.824
A549	TPOT	0.960	0.914	0.937	0.856	0.937	0.732	0.851	0.792	0.585	0.792
Consensus		NA	NA	NA	NA	NA	0.786	0.893	0.840	0.684	0.885

**Table 4:** Comparison of the predictive performance of the consensus model using transcriptomics data for the ER and AR binding endpoints on the validation set against the predictive performance of QSAR models for the same endpoints of the CERAPP (Collaborative Estrogen Receptor Activity Prediction Project) and CoMPARA (Collaborative Modeling Project for Androgen Receptor Activity) models, respectively.

Endpoint	Dataset	Model	Sensitivity	Specificity	Balanced Accuracy
ER binding	Validation set (All)	CERAPP	0.35	0.879	0.615
		QGexAR consensus	0.570	0.880	0.725
	Validation set (CERAPP high confidence > 1 source)	CERAPP	0.490	0.883	0.687
		QGexAR consensus	0.705	0.905	0.806
AR binding	Validation set (All)	CoMPARA	0.911	0.645	0.778
		QGexAR consensus	0.622	0.903	0.762
	Validation set (high confidence > 1 source)	CoMPARA	0.882	0.643	0.763
		QGexAR consensus	0.706	0.893	0.799

**Table 5:** Predictive performances for ER binding, AR binding, and TR antagonist in the presence of an applicability domain based on cosine similarity. Predictive performance is evaluated on the validation set shared by the five cell lines. Random Forest (RF) and Support Vector Machine (SVM).

Cell Line	Best Cross Validation Algorithm	Cosine Similarity Threshold	Sensitivity	Specificity	Balanced Accuracy	MCC	AUC	Coverage (%)
ER binding								
MCF7	rf	0.348	0.672	0.807	0.739	0.480	0.739	60.5
A549	rf	0.319	0.672	0.758	0.715	0.427	0.715	51.2
AR binding								
MCF7	svm	0.340	0.821	0.654	0.738	0.417	0.738	54.5
A549	svm	0.302	0.875	0.736	0.806	0.537	0.806	48.0
TR antagonist								
MCF7	tpot	0.347	0.846	0.845	0.845	0.684	0.845	64.7
A549	tpot	0.310	0.824	0.800	0.812	0.619	0.812	52.7

## Data Availability

The raw data supporting the conclusions of this article will be made available by the authors on request.

## Supplementary Materials

The following supporting information can be downloaded at: https://www.mdpi.com/article/10.3390/toxics12080541/s1. Figure S1: UMAP of the transcriptomics profiles for each chemical in each dataset for the 2 cell lines; Figure S2: Umap made on z-score for each gene category on each dataset considering all cell lines; Table S1: Table summarizing the different parameters manually set in the different models. Other parameters not shown were default parameters of the method; Table S2: Performance table for the different endpoint dataset with the different protocol tested in 10 iteration of 5 fold cross validation; Table S3: Number of descriptors retained after feature selection and the number of which showed significant difference between active and inactive compounds for the different endpoints.
