# Supplementary material for: Prediction of Endocrine-Disrupting Chemicals Related to Estrogen, Androgen, and Thyroid Hormone (EAT) Modalities Using Transcriptomics Data and Machine Learning
Source: Toxics. 2024 Jul 26;12(8):541. doi: 10.3390/toxics12080541 (PMC11360171; doi:10.3390/toxics12080541)
Supplement: Supplementary file 1 [file toxics-12-00541-s001.zip › toxics-3092492-supplementary.pdf]

Supplementary Materials

Table S1. Table summarizing the different parameters manually set in the different models. Other parameters not shown were default parameters of the method. * parameters optimized using grid search with 5 fold cross-validation and by adopting the balanced accuracy scoring method.

Model	Features operation	Hyperparameters	Values
catboost		learning_rate*	0.1
		depth*	4,6
		l2_leaf_reg*	1,3,5
		loss_function	Logloss
		auto_class_weights	Balanced
xgboost		min_child_weight*	1, 5, 10
		gamma*	0.5, 1, 1.5, 2, 5
		subsample*	0.6, 0.8, 1.0
		colsample_bytree*	0.6, 0.8, 1.0
		max_depth*	3, 4, 5
		learning_rate	0.1
		n_estimators	100
		max_delta_step*	0,1
		objective	binary:logistic
		scoring	balanced_accuracy
		scale_pos_weight	number_active/number_inactive
svm_SGDClassifier	Standardisation	average*	True, False
		l1_ratio*	0., 0.111, 0.222, 0.333, 0.444,0.555, 0.666, 0.777, 0.888, 1.
		alpha*	0.01,0.1 ,1
		loss	hinge
		penalty	elasticnet
		class_weight	balanced
reglog_elasticnet	Standardisation	max_iter*	1000, 10000
		l1_ratio*	0., 0.111, 0.222, 0.333, 0.444,0.555, 0.666, 0.777, 0.888, 1.
		penalty	elasticnet
		class_weight	balanced
		solver	saga
random_forest		min_samples_leaf*	1, 2, 3, 4, 5, 6, 7, 8, 9, 10
		max_depth*	None,1, 2, 3, 4, 5, 6, 7, 8, 9, 10
		min_samples_split*	2, 5, 10
		class_weight	balanced
AutoKeras		epochs	100
		max_trials	50
tpot		generations	5
		population_size	50
		scoring	balanced_accuracy

deep learning models: dnn_1, dnn_2, dnn_3, dnn_4	monitor	acc
	patience	4
	min_delta	0.001
	mode	max
	optimizer	adam
	loss	binary_crossentropy
	epochs	200
	batch_size	32

* Hyperparameters optimized with grid search

Table S2. Performance table for the different endpoint dataset with the different protocol tested in 10 iteration of 5 fold cross validation. In bold the best performance of mean balance accuracy across the protocols.

Features / CELL LINES	Algorithm	# Features	Mean sensitivity	Std sensitivity	Mean specficity	Std specficity	Mean Balanced accuracy	Std Balanced accuracy	Mean MCC	Std MCC	Mean AUC	Std AUC
**ER binding**												
All descriptors + features selection												
MCF7	xgboost	2117	0.447	0.051	0.853	0.024	0.650	0.029	0.325	0.061	0.714	0.029
A549	xgboost	2099	0.397	0.069	0.821	0.033	0.609	0.038	0.236	0.081	0.667	0.035
Landmark + Best inferred descriptors + features selection												
MCF7	xgboost	1798	0.426	0.041	0.876	0.024	0.651	0.024	0.338	0.052	0.717	0.031
A549	xgboost	1850	0.418	0.065	0.815	0.031	0.616	0.037	0.249	0.078	0.667	0.036
Landmark + features selection												
MCF7	rf	70	0.482	0.050	0.863	0.024	**0.672**	0.029	0.370	0.061	0.748	0.031
A549	rf	80	0.413	0.057	0.845	0.032	**0.629**	0.032	0.286	0.069	0.712	0.034
Landmark												
MCF7	xgboost	978	0.433	0.056	0.855	0.027	0.644	0.031	0.315	0.066	0.701	0.027
A549	xgboost	978	0.410	0.058	0.809	0.038	0.609	0.034	0.235	0.072	0.657	0.040
**AR binding**												
All descriptors + features selection												
MCF7	dnn_4	2050	0.496	0.087	0.900	0.030	0.698	0.038	0.421	0.068	0.785	0.041
A549	dnn_4	1553	0.547	0.089	0.870	0.045	0.709	0.039	0.429	0.072	0.766	0.052
Landmark + Best inferred descriptors + features selection												
MCF7	svm	1770	0.614	0.133	0.810	0.086	0.712	0.045	0.396	0.078	0.712	0.045
A549	svm	1391	0.661	0.074	0.765	0.060	0.713	0.038	0.391	0.074	0.713	0.038
Landmark + features selection												
MCF7	svm	72	0.676	0.079	0.754	0.045	**0.715**	0.039	0.374	0.068	0.715	0.039
A549	svm	73	0.719	0.083	0.796	0.049	**0.757**	0.047	0.473	0.088	0.757	0.047
Landmark												
MCF7	elasticnet	978	0.515	0.089	0.859	0.029	0.687	0.043	0.369	0.077	0.758	0.045
A549	elasticnet	978	0.604	0.079	0.866	0.036	0.735	0.041	0.469	0.078	0.792	0.041

**TR antagonist**											
All descriptors + features selection											
MCF7	tpot	2486	0.763	0.053	0.806	0.042	0.784	0.032	0.550	0.062	0.841	0.030
A549	tpot	2191	0.556	0.058	0.935	0.027	0.745	0.032	0.551	0.066	0.816	0.035
Landmark + Best inferred descriptors + features selection												
MCF7	xgboost	2079	0.641	0.065	0.900	0.022	0.771	0.033	0.568	0.059	0.852	0.030
A549	xgboost	1897	0.611	0.063	0.897	0.028	0.754	0.033	0.540	0.063	0.825	0.033
Landmark + features selection												
MCF7	tpot	77	0.701	0.055	0.873	0.025	0.787	0.031	0.579	0.059	0.857	0.027
A549	tpot	76	0.725	0.070	0.821	0.045	**0.773**	0.033	0.542	0.065	0.811	0.034
Landmark												
MCF7	tpot	978	0.733	0.056	0.846	0.031	**0.789**	0.028	0.572	0.053	0.838	0.030
A549	tpot	978	0.564	0.052	0.931	0.025	0.748	0.024	0.553	0.045	0.820	0.033

Table S3. Number of descriptors retained after feature selection and the number of which showed significant difference between active and inactive compounds for the different endpoints.

Endpoint	Descriptors	MCF7	A549
ER binding	# All features	12328	12328
	# All features with p-value < 0.05	4812 (47.30%)	3267 (32.11%)
	# landmark + multisurf	70	80
	# landmark + multisurf with p-value < 0.05	53 (75.71%)	44 (55.00%)
AR binding	# All features	12328	12328
	# All features with p-value < 0.05	3315 (32.58%)	4507 (44.30%)
	# landmark + multisurf	72	73
	# landmark + multisurf with p-value < 0.05	37 (51.39%)	57 (78.08%)
TR antagonist	# All features	12328	12328
	# All features with p-value < 0.05	5474 (53.80%)	4257 (41.84%)
	# landmark + multisurf	77	76
	# landmark + multisurf with p-value < 0.05	70 (90.91%)	69 (90.79%)

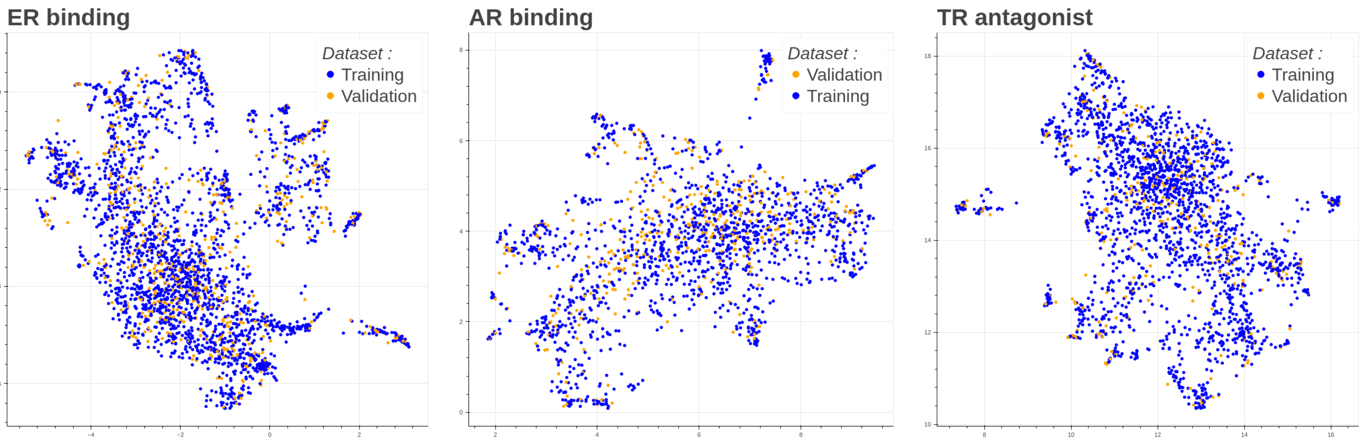

(a) (b) (c)

Figure S1: UMAP of the transcriptomics profiles for each chemical in each dataset for the 2 cell lines. Each dot corresponds to a chemical. Blue dots correspond to chemicals of the training set and orange dots correspond to chemicals of the validation set. Only the landmark genes were considered. (a) Profile for the ER binding dataset. (b) Profile for the AR binding dataset. (c) Profile for the TR antagonist dataset.

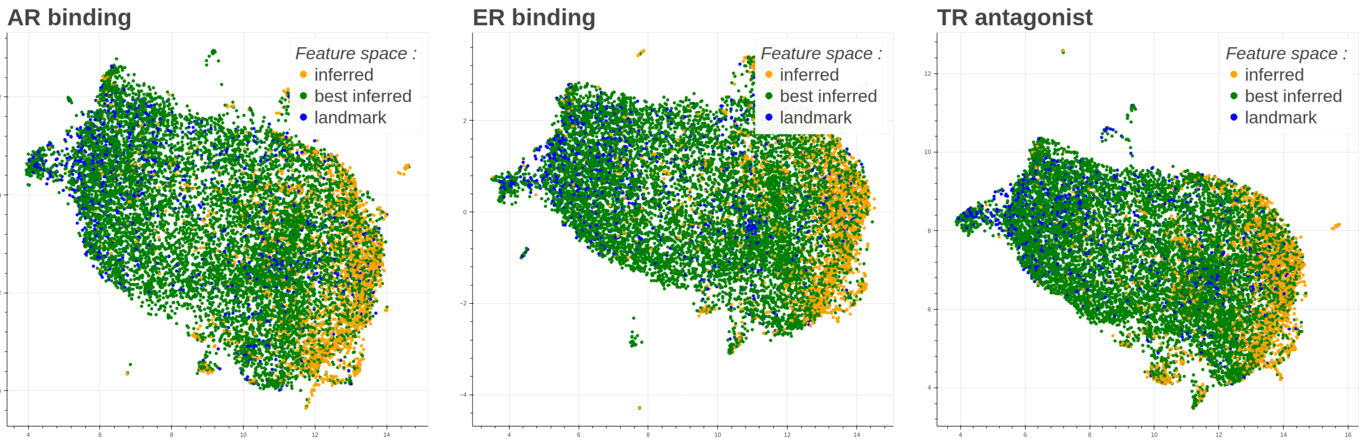

(a) (b) (c)

Figure S2: UMAP made on z-score for each gene category on each dataset considering all cell lines. (a) Profile for the ER binding dataset. (b) Profile for the AR binding dataset. (c) Profile for the TR antagonist dataset.
